# Recent Progress in Electrochemical Biosensors for Glycoproteins

**DOI:** 10.3390/s16122045

**Published:** 2016-12-01

**Authors:** Uichi Akiba, Jun-ichi Anzai

**Affiliations:** 1 Graduate School of Engineering and Science, Akita University, 1-1 Tegatagaluenn-machi, Akita 010-8502, Japan; uakiba@gipc.akita-u.ac.jp; 2 Graduate School of Pharmaceutical Sciences, Tohoku University, Aoba, Aramakim, Sendai 980-8578, Japan

**Keywords:** biosensor, electrochemical sensor, glycoprotein, immunosensor, lectin, phenylboronic acid, molecularly imprinted polymers

## Abstract

This review provides an overview of recent progress in the development of electrochemical biosensors for glycoproteins. Electrochemical glycoprotein sensors are constructed by combining metal and carbon electrodes with glycoprotein-selective binding elements including antibodies, lectin, phenylboronic acid and molecularly imprinted polymers. A recent trend in the preparation of glycoprotein sensors is the successful use of nanomaterials such as graphene, carbon nanotube, and metal nanoparticles. These nanomaterials are extremely useful for improving the sensitivity of glycoprotein sensors. This review focuses mainly on the protocols for the preparation of glycoprotein sensors and the materials used. Recent improvements in glycoprotein sensors are discussed by grouping the sensors into several categories based on the materials used as recognition elements.

## 1. Introduction

Electrochemical biosensors are fabricated by modifying the surfaces of electrodes with biomaterials such as proteins and DNA that selectively bind target compounds [1,2,3,4]. The specific binding or interactions between biomaterials and the target compound on the electrode surface are then converted to changes in electrical current, electrode potential, and/or impedance as the output signals of the biosensors. Thus, electrochemical biosensors can be used to determine target compounds in biological fluids such as blood without pre-treatment of samples. Consequently, a variety of compounds have been analyzed via electrochemical biosensors in laboratories and hospitals. Glucose biosensors, which are widely used for detecting glucose in blood for the diagnosis and treatment of diabetic patients, represent a typical example of an electrochemical biosensor [5,6]. Glucose biosensors are available commercially. 

Recently, much attention has been focused on the development of electrochemical biosensors that are sensitive to biomarkers produced and secreted from cells and tissues as a result of diseases and disorders. Many proteins have been identified as tumor markers produced at higher levels in cancerous conditions. For example, carcinoembryonic antigen (CEA), and α-fetoprotein (AFP) are recognized as typical biomarkers for cancers [7]. In addition, biosensors for detecting biomarkers of infectious disease and life style-related diseases have been extensively studied. Glycated hemoglobin (HbA1c) is a typical biomarker that increases as a result of hyperglycemia in diabetic patients [8]. It is worth noting that biomarker proteins often contain hydrocarbon chains on the surfaces (i.e., glycoproteins), for example, CEA, AFP, and HbA1c. In this context, a summary of the recent progress made in the study of glycoprotein biosensors would be of benefit to researchers and engineers working in the field of biomedical analysis. 

**Figure 1:**
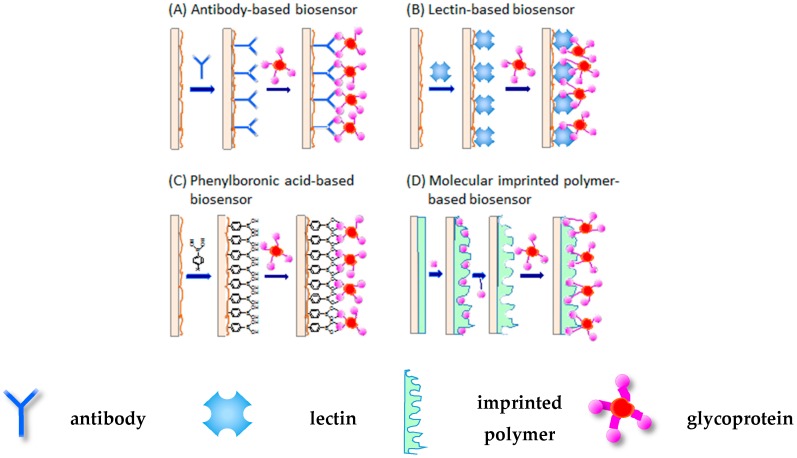
Different types of glycoprotein biosensors based on (**A**) antibody; (**B**) lectin; (**C**) phenylboronic acid; and (**D**) molecular imprinted polymer.

Glycoprotein sensors can be divided into several categories based on the type of recognition elements used. The recognition elements are immobilized on the surfaces of biosensors to form selective binding sites for glycoproteins. The glycoprotein sensors in the first category are constructed using anti-glycoprotein antibody as a recognition element (Figure 1A). This is a straightforward route for the construction of glycoprotein sensors because specific antibodies to certain proteins can be prepared by established procedures, or are available commercially. The second category of glycoprotein sensors utilizes lectins, a family of sugar-binding proteins, as the recognition element (Figure 1B). Concanavalin A (Con A) is a typical lectin protein that is predominantly used for fabricating biosensors. Con A can be used to immobilize polysaccharides and glycoproteins onto solid supports because it contains binding sites to D-glucose and D-mannose residues. A variety of lectins are now commercially available. The third category of sensor relies on phenylboronic acid (PBA) derivatives (Figure 1C). PBA and its derivatives are known to bind 1,2- and 1,3-diol compounds such as sugars by forming boronate ester bonds. Because of their selective binding to sugars, PBAs are sometimes called synthetic lectins. Consequently, PBA-modified electrodes are able to selectively bind glycoproteins as in the cases of antibody- and lectin-modified sensors. An advantage of using PBAs for glycoprotein sensor construction is that their chemical structures can be suitably designed and synthesized. Molecularly imprinted polymers (MIP) are another platform used to construct glycoprotein sensors (Figure 1D), although the number of examples of these sensors is limited. MIPs are prepared by polymerization of monomers in the presence of target molecules, followed by removal of the targets from the resulting polymers. This process provides MIPs that contain cavities with complementary shapes and sizes for the targets. Among the four types of sensors, antibody- and lectin-based sensors usually exhibit high selectivity to target molecules owing to a high specificity of the proteins to targets. In contrast, the selectivity of PBA- and MIP-based biosensors is not always satisfactory because these elements are of synthetic origin. The synthesis of PBAs and MIPs with high selectivity to their targets is still a challenge in the development of high-performance glycoprotein sensors. 

This review deals with not only glycoproteins but also related compounds such as erythrocytes and leukemia cells which contain carbohydrate chains on the surface. In addition, biosensors for viruses and bacteria are also discussed based on their specific interactions with carbohydrates. 

Several papers have reviewed the preparation of glycoprotein sensors and their use in biomedical analysis [9,10,11,12,13]. A variety of glycoprotein sensors with different detection modes including colorimetric, fluorometric, gravimetric, and electrochemical techniques have been reported so far. This article reviews recent progress made in the development of electrochemical glycoprotein sensors, focusing on the literature published over the last several years. In the following section, we begin with an overview of electrochemical glycoprotein sensors prepared using antibodies as the recognition element (i.e., immunosensors). 

## 2. Immunosensors for Glycoproteins

Electrochemical immunosensors, constructed by immobilizing antibodies on the surface of electrodes, exhibit high sensitivity to target compounds owing to the specific binding of antibody to target. According to this strategy, various kinds of glycoprotein sensors have been prepared. An important issue in the development of high-performance immunosensors is to enhance the detection sensitivity. This is especially true for the detection of biomarker proteins because the concentration of biomarkers in biological fluids is usually low. Thus, it is necessary to selectively detect biomarker proteins in biological samples. To achieve this goal, a variety of strategies have been employed as discussed below.

### 2.1. Functional Polymer-Based Sensors

Functional polymers have been widely used to modify the surface of electrode in constructing biosensors. A variety of commercially available polymers are utilized for this purpose. In addition, it is possible to synthesize polymers with functional moieties including ionic groups, hydrophobic/hydrophilic groups, reactive groups, and so forth. Thus, functional polymers can be used for immobilizing proteins on the electrode surface, reducing interference, and enhancing the stability of biosensors. 

Bhatti and coworkers have prepared immunosensors for prostate-specific antigen (PSA), a biomarker for prostate cancer, using nanostructured (NS) gold (Au) electrodes characterized by polymer brush-modified surfaces [14]. The polymer brush of poly(methacrylic acid) bearing glycidyl side chains was synthesized on the NS Au electrode by surface-initiated atom transfer radical polymerization. A large amount of anti-PSA antibodies are covalently immobilized to the polymer brush through the glycidyl moieties. After binding the target PSA on the electrode, a silica nanoparticles-labeled secondary antibody is deposited on the electrode to enhance the output signal. In fact, PSA sensors based on the NS Au electrode show higher responses to PSA than NS-free Au electrode sensors in impedimetric and voltammetric measurements. Typically, PSA sensors based on NS Au electrodes show a dynamic range over PSA concentrations of 0.005–1000 ng·mL^−1^ with a lower detection limit of 2.3 pg·mL^−1^, while those of NS-free sensors are 0.03–1000 ng·mL^−1^ and 10 pg·mL^−1^, respectively. In another attempt to enhance the sensitivity of PSA immunosensors, β-cyclodextrin (β-CD) monolayer-coated Au electrodes were modified with anti-PSA antibody, in which β-CD afforded gates for the electron transfer of redox probes [15]. β-CD is a cyclic oligo-saccharide that exhibits binding affinity to small molecules [16]. The voltammetric current of the sensor was recorded in the solution containing Fe(CN)_6_^3−/4−^ ions, where the response current decreases in the presence of PSA due to the steric hindrance for the electron transfer of the Fe(CN)_6_^3−/4−^ ions. The immunosensor exhibits a linear response to PSA in the range from 0.001–1.0 ng·mL^−1^ with a lower detection limit of 0.3 pg·mL^−1^. 

### 2.2. Carbon Nanomaterial-Based Sensors

**Figure 2:**
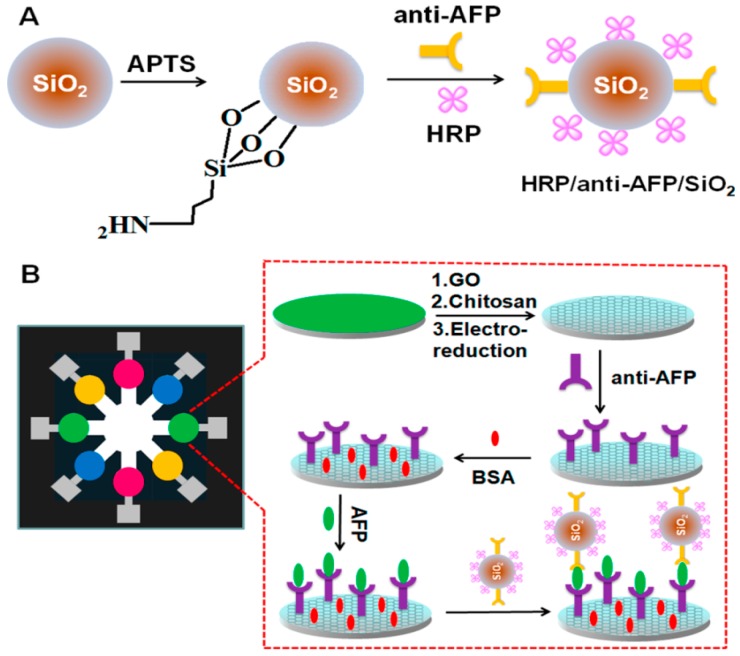
(**A**) Immobilization of anti-AFP on SiO_2_ microparticles; (**B**) Microfluidic-based immunosensors for AFP. Reprinted with permission from Wu et al. [25].

Recently, much attention has been devoted to the use of graphene in the development of electrochemical sensors because of its high surface area and excellent conductivity [17]. Amperometric immunosensors for thrombomodulin (TM) have been constructed using an Au electrode modified with graphene and silver/silver oxide (Ag-Ag_2_O) particles [18]. TM is an endothelial glycoprotein found at higher levels in blood in association with endothelial cell injury and the progression of autoimmune disorders. The composite-deposited Au electrode is further modified with anti-TM antibody to bind TM. The redox current of the sensor, which originates from the redox reactions of Ag-Ag_2_O nanoparticles, decreases depending on the concentration of TM in the range of 0.1–20 ng·mL^−1^ at pH 7.4. Thus, the graphene/Ag-Ag_2_O nanocomposite is useful as an electrode modifier for constructing TM immunosensors. The usefulness of graphene-modified electrodes in immunosensor applications has been further demonstrated. An epitaxial grown graphene film was modified with human chorionic gonadotropin antibody (anti-hCG) for the construction of hCG sensors [19]. hCG is a diagnostic biomarker of pregnancy as well as of cancerous tumors in the ovaries and testes. The electrical resistance of the hCG sensor linearly changes in response to hCG in the range of 0.62–5.62 ng·mL^−1^ with a lower detection limit of 0.62 ng·mL^−1^. A comparison of the performance of the sensor with that of standard enzyme-linked immunosorbent assay (ELISA) showed that the lower detection limit of the sensors is 30-times more sensitive than that of ELISA. Immunosensors for carbohydrate antigen 153 (CA153), a biomarker for breast cancer, have also been constructed using graphene-modified electrodes. Turner and coworkers prepared CA153 sensors by depositing antibody-modified graphene sheets on a glassy carbon electrode [20]. The voltammetric current of the sensor recorded in the Fe(CN)_6_^3−/4−^ solutions changes depending on the concentration of CA153 in the range of 0.1–20 U mL^−1^. In addition, CA153 immunosensors based on graphene-modified electrodes coupled with Cd^2+^ ion-functionalized porous TiO_2_ have been reported [21]. CA153 sensors exhibit voltammetric signals originating from the redox reactions of the Cd^2+^ ion. The sensors show a linear response to 0.02–60 U mL^−1^ of CA153 with a 0.008 U mL^−1^ detection limit. Graphene can be further functionalized by coupling with Au-Ag nanocomposites or Au-Prussian blue composites for immunosensor applications [22,23]. It has also been reported that graphene-coated microfluidic sensors are highly sensitive to cancer biomarkers including CEA, AFP, CA153, and cancer antigen 125 (CA125) (Figure 2) [24,25]. A recent paper reports a novel approach to increase the degree of miniaturization and sensitivity of biosensor platforms by microfluidic stop-flow techniques [26]. 

Carbon nanotubes (CNTs) are another useful option as carbon nanomaterials for improving the performance of biosensors [27,28,29]. hCG sensors have been constructed by using single-wall CNT-based screen-printed electrodes modified with anti-hCG antibody [30]. The surface of CNTs is first functionalized with amino-terminated silane coupling agent and then anti-hCG antibody is covalently attached. The hCG sensor thus prepared shows a linear response in electrochemical impedance analysis to hCG in the concentration range 0.01–100 ng·mL^−1^. This sensor can be used for detecting hCG in urine samples from pregnant women. Multi-wall CNTs have also been used as a component of biosensors that detect cell surface glycan expression as cancer biomarkers [31].

### 2.3. Metal Nanoparticle-Based Sensors

Metal nanoparticles have been used for improving the performance of biosensors owing to their excellent properties, including high conductivity, high surface area-to-weight ratio, and easy modification using synthetic and biological molecules [32]. Au nanoparticle-labeled antibodies can be used as catalysts in electrochemical stripping analysis. Au nanoparticle-based immunosensors are prepared by modifying the surface of a screen-printed electrode with anti-human cytomegalovirus glycoprotein B (anti-human cytomegalovirus (HCMV) glycoprotein B) [33]. The antibody-modified electrode is further incubated in solutions of HCMV glycoprotein B and anti-HCMV glycoprotein B labeled with Au nanoparticles. The Au nanoparticles serve as catalysts for the reductive deposition of Ag nanoparticles on the electrode surface. The detection of HCMV glycoprotein B is conducted through electrochemical stripping analysis of the Ag nanoparticles deposited. Thus, the sensors show lower detection limits of 3.3 ± 1.7 ng·mL^−1^ in urine samples. On the other hand, 4-mercaptobenzoic acid (4MBA)-modified Au nanoparticles have also been used to construct immunosensors for dengue virus [34]. The 4MBA-modified Au nanoparticles serve as a conductive bridge that links the antibody and electrode. The immunosensors can be used to detect four kinds of dengue virus serotypes based on impedimetric and voltammetric measurements. Au nanoparticles combined with CdSe quantum dots and with horseradish peroxidase (HRP) have been successfully used to prepare CA125 and AFP sensors, respectively [35,36]. Ag nanoparticle-containing nanocomposites are also useful as components of electrochemical biosensors. Ag nanoparticles/glucose oxidase (GOx) nanocomposite has been used to construct CEA immunosensors [37]. The sensor exhibits a voltammetric response to 0.001–50 ng·mL^−1^ of CEA upon adding glucose to the sample solutions. Another example includes PSA sensors equipped with Ag nanoparticles/zinc oxide nanocomposites [38]. The sensors show a linear response to PSA in the concentration range 0.004–60 ng·mL^−1^ with a detection limit of 1.5 pg·mL^−1^. 

**Figure 3:**
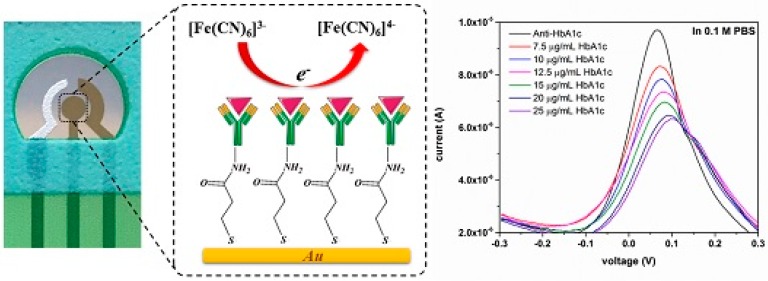
Response mechanism of label-free immunosensor for HbA1c sensor and its DPV in the presence of HbA1c. Reprinted from Liu et al. [41].

Glycated hemoglobin (HbA1c) level in blood is an indicator that reflects the average glucose level over the preceding 2–3 months. A normal HbA1c level is in the range of 4%–6%. Thus, a variety of techniques have been developed to determine HbA1c levels [39,40]. Electrochemical HbA1c sensors have also been studied for the development of rapid and simple protocols for detecting HbA1c levels. For example, single-use disposable HbA1c sensors have recently been developed using micro-fabrication techniques including sputtering, vapor deposition, and thick-film printing (Figure 3) [41]. The voltammetric response of the disposable sensors is recorded in the sample solutions containing Fe(CN)_6_^3−/4−^ ions as a redox indicator. The redox current of the sensor decreases with increasing concentrations of HbA1c due to the suppressed access of Fe(CN)_6_^3−/4−^ ions to the electrode surface. The sensor shows linear responses to 7.50–20 µg·mL^−1^ of HbA1c in buffer solution and 0.1–0.25 mg mL^−1^ of HbA1c in undiluted human serum. 

In another work, Au nanoparticles-bridged ferrocene (Fc) derivatives bearing a glycosylated pentapeptide as an epitope that can bind anti-HbA1c antibody have been used as components of HbA1c sensors [42,43]. Binding of anti-HbA1c to the epitope on the electrode surface results in suppressed redox reactions of the Fc moiety of the sensor. Thus, the output current of the sensors depends on the concentration of HbA1c in sample solutions containing anti-HbA1c in a competitive binding assay. The redox current increases linearly with increasing concentrations of HbA1c from 4.6% to 15.1%, which includes normal and diabetic HbA1c levels.

### 2.4. FET Sensors

Electrochemical biosensors are usually constructed using metal or carbon electrodes for amperometric, voltammetric, or impedimetric measurements as described above. An ion-sensitive field effect transistor (ISFET) is another device available for the construction of potentiometric biosensors. ISFET was first developed as a potentiometric sensor sensitive to H^+^ ions. The sensing principle of ISFET relies on changes in gate potential of the device originating from the binding of H^+^ ions. Since then, a variety of biosensors have been prepared using ISFET coupled with enzymes, antibodies, and so forth [44,45]. ISFET can be used to detect other charged molecules such as proteins. Based on this strategy, FET devices have been utilized for the construction of glycoprotein sensors. A bovine herpes virus-1 (BHV-1) sensor has been prepared using metal–oxide–semiconductor FET (MOSFET) as a transducer coupled with an Au chip [46]. The Au chip is modified with anti-BHV-1 antibody and electrically connected to the gate terminal of MOSFET. The FET sensors thus prepared show a potentiometric response to BHV-1 depending on the dilution of BHV-1 serum. The sensors achieve comparable performance to other methods such as surface plasmon resonance sensors and ELISA. The authors claim that the FET sensors show promise as point-of-care devices for the diagnosis of serological diseases. Polypyrrole (PPy) nanotubes-modified FET biosensors have also been constructed [47]. The PPy nanotubes are first covalently modified with an aptamer sensitive to CEA and deposited on an interdigitated array electrode. The PPy nanotube-based FET sensors thus prepared exhibit a rapid response (<1 s) to CEA with a lower detection limit of 1 fg·mL^−1^. The detection limit is 2–3 orders of magnitude more sensitive than that reported for other CEA sensors. 

FET immunosensors sensitive to HbA1c have been developed [48,49,50]. These FET sensors are prepared using Au nanoparticles to enhance the surface area of the extended gate. The Au nanoparticles-deposited gate is further modified with anti-HbA1c or anti-Hb antibody to afford FET sensors sensitive to HbA1c or Hb, respectively. The sensors exhibit potentiometric responses to 4–24 µg·mL^−1^ of HbA1c and 60–180 µg·mL^−1^ of Hb. 

As discussed above, FET-based glycoprotein sensors detect changes in the surface potential at the gate surface associated with specific binding of analytes. Therefore, one should carefully design the sensors as well as sample preparation by keeping in mind that potentiometric response of FETs strongly depends on the ionic strength of media because Debye length at the surface/solution interface shrinks in the presence of high concentration of ions. 

## 3. Lectin-Based Sensors for Glycoproteins

Lectins are a family of sugar-binding proteins present in various sources including plants, bacteria, and animals [51,52]. Lectins have been widely used to immobilize sugar-tagged biomolecules onto solid supports because they selectively bind carbohydrate chains of glycoproteins and glycolipids [53,54]. For instance, GOx and HRP can be immobilized on the surface of an electrode through lectin complexation because GOx and HRP intrinsically contain hydrocarbon chains on the surfaces. The GOx and HRP-modified electrodes thus prepared are used as enzyme biosensors [55,56,57,58,59]. Con A is the most widely studied lectin protein, and is known to contain sugar-binding sites selective to D-mannose and D-glucose. The binding constants of Con A to D-mannose and D-glucose are reported to be 2.2 × 10^3^ and 0.8 × 10^3^ M^−1^, respectively [60]. Lectins bind glycoproteins and other biomaterials rather strongly through multivalent bindings despite the relatively low binding constants of lectins for each sugar. Various lectin proteins with different selectivity levels are available commercially. It is worth noting that lectin proteins have been employed as materials for the construction of three-dimensional protein architectures on solid surfaces in the development of biosensors [61,62,63,64]. The following sections discuss lectin-based glycoprotein sensors constructed on self-assembled monolayer (SAM) films and polymer films, as well as metal nanoparticles- and phenylboronic acid-based sensors.

### 3.1. SAM-Based Sensors

SAM-modified electrodes have widely been utilized for the construction of electrochemical biosensors because of their versatility in chemical structures and properties. A common protocol for the formation of SAMs on electrodes relies on covalent bonding between thiol derivatives and Au electrodes [65,66]. Glycoprotein sensors based on lectin-modified electrodes are prepared by covalently attaching lectin to the surface of SAM-modified Au electrodes, as discussed below. For this purpose, Au electrodes are coated with mixed SAMs comprising carboxyl-terminated long-chain thiols, followed by covalent bonding with the amine groups of lectins. Covalent immobilization of lectins affords a stable layer for using the modified electrodes as biosensors. 

**Figure 4:**
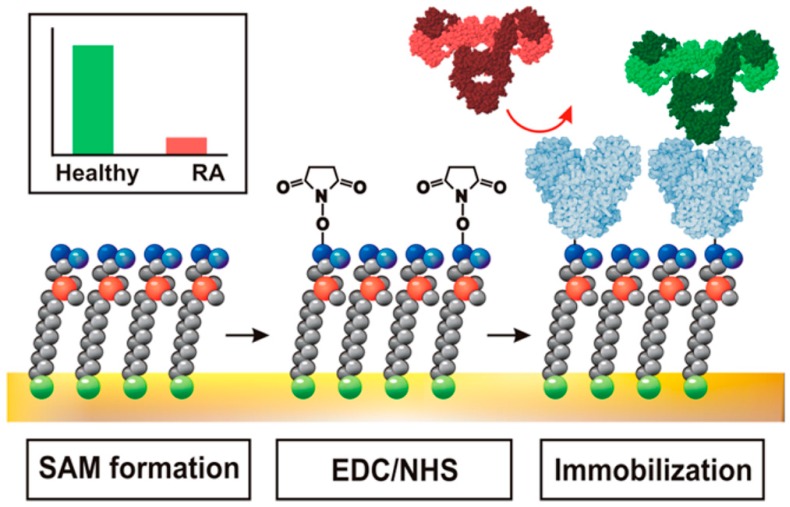
Preparation of carboxybetaine SAM-based SNA sensors for glycoprofiling. Reprinted from Bertok et al. [69]. SNA sensor was prepared by initial formation of carboxybetaine SAM on Au electrode, followed by conversion of carboxyl groups to NHS active esters and finally covalent attachment of SNA.

According to this protocol, various lectins including *Sambuccus nigra* agglutinin (SNA), *Maackia amurensis* agglutinin, and *Ricinus communis* agglutinin (RCA) have been covalently immobilized on carboxyl-terminated SAM-modified electrodes to prepare glycoprotein sensors [67,68,69,70]. The SNA-immobilized sensors show ultrasensitive detection limits as low as 0.33 fM for fetuin and 0.54 fM for asialofetuin in impedimetric measurements [67]. It is also possible to use the SNA-immobilized sensors for detecting changes in the fraction of sialic acid on fetuin. Mixed SAMs composed of 11-mercaptoundecanoic acid and sulfobetaine-terminated thiol can effectively suppress the nonspecific adsorption of proteins [68]. Sensors prepared using sulfobetaine-containing SAM show impedimetric response to glycoproteins such as invertase, transferrin, fetuin, and asialofetuin down to femtomolar levels. Carboxybetaine thiols are also effective for the preparation of SAMs to which lectins can be covalently immobilized. Impedimetric sensors prepared using carboxybetaine SAM with SNA have been used for glycoprofiling of antibodies isolated from the human sera of rheumatoid arthritis patients (RA) and healthy subjects (Figure 4) [69]. 

The antibodies from rheumatoid arthritis patients are discriminated from those of healthy subjects based on changes in the amount of sialic acid residues in the antibodies. It was demonstrated using RCA-based impedimetric sensors that changes in the glycan structure of antibodies isolated from human serum are closely associated with rheumatoid arthritis [70]. It is possible to use SAM-coated Au electrodes modified with an oligosaccharide to detect lectin and influenza hemagglutinin [71,72]. Bueno and coworkers have prepared lectin-immobilized sensors for studying lectin-glycoprotein interactions based on the impedimetric and capacitive responses of the sensors [73,74]. For this purpose, ArtinM lectin was covalently immobilized on a SAM-coated electrode and the binding behavior to HRP and glycoprotein-bearing leukemia cells was studied. 

A variety of materials are available for preparing SAMs on electrode surfaces for the construction of electrochemical sensors. Thiolated triethyleneglycols bearing α-mannose and β-galactose residues have been used to prepare SAMs on Au electrodes for the construction of impedimetric sensors for *E. coli* ORN-178 [75]. It is known that ORN-178 strain of *E. coli* exhibits specific binding to α-mannose residue, whereas the ORN-208 strain does not show α-mannose-specific binding [76]. The α-mannose-modified sensor exhibits responses to *E. coli* in a range of bacterial concentrations in 10^2^–10^3^ colony-forming units (cfu) mL^−1^, while no response is observed for *E. coli* ORN-208 because of a lack of affinity to α-mannose. On the other hand, the surface of boron-doped diamond (BDD) electrode has been modified using SAM with carbohydrate residues [77]. The hydroxyl groups on an oxidized BDD electrode are first modified with an alkynyl-substituted pentanoic acid, followed by copper-catalyzed click reaction with a carbohydrate derivative with azide functionality. The sensors thus prepared show impedimetric response to *Lens culinaris* lectin with a detection limit of 5 ± 0.5 nM. A similar protocol for the formation of sialic acid SAMs on BDD electrodes has been reported, in which ethynylbenzene is first introduced on the BDD surface and, after deprotection, sialic acid-mimic peptide terminated with an azide group is immobilized *via* click reaction [78]. The BDD electrode is used for detecting influenza virus in the range of 20–500 plaque-forming units (pfu), based on the high affinity of influenza virus to sialic acid [79]. BBD electrode is known for its excellent characteristics including its wide potential window and negligible nonspecific adsorption of biomolecules [80]. The surface modification of silicone substrates has also been carried out using bifunctional oligoethyleneglycol with an azido group, to which sugars are introduced by click reaction [81]. 

### 3.2. Polymer Film-Based Sensors

The use of polymer films is another choice for immobilizing lectins on electrodes. Polymer films provide a three-dimensional scaffold for lectin binding, in which lectins can be immobilized through multivalent bonding in contrast to the two-dimensional surface of SAMs. Thus, stable biointerfaces can be constructed using polymer films. Of special interest is the use of conducting polymers because their electrical properties, such as conductivity and resistance, can be employed as output signals of the sensors without the addition of redox labels or reagents. Various conducting polymers have been developed so far and some are available commercially. 

Polyaniline bearing mannose residues (man-PANI) have been used for the construction of man-PANI-coated electrodes to study the impedimetric response of the electrodes upon binding Con A [82]. X-ray photoelectron and UV-visible absorption spectroscopic studies revealed that Con A binding to man-PANI films induces the conversion of amine functionality in the PANI backbone into the imine form. Accordingly, the impedimetric signal of the sensor changes upon Con A binding, enabling quantitative determination of Con A down to 0.12 nM. Quinone-fused polythiophene polymers bearing carbohydrate moieties were coated on an electrode surface to further immobilize Con A, which binds *E. coli* through specific binding of lipopolysaccharide [83]. *E. coli* is detected by the polythiophene-coated electrodes with a detection limit of 25 cells mL^−1^. An interesting study was carried out using the Con A-modified electrodes for an assay of antibiotic activity [84]. Antibiotics ciprofloxacin, ceftriaxone, and tetracycline were incubated with *E. coli* 1485 for 18 h and the responses of Con A-modified electrode to the *E. coli* 1485 were measured before and after incubation. The results showed that the responses of the sensor to *E. coli 1485* reduce to 23%, 27%, and 38% of the original signals in the presence of the antibiotics, respectively. This protocol may be useful for therapeutic management of the drugs. 

A polymer brush can be prepared by grafting polymer chains from the surface to form a polymer layer with defined thickness. It is envisaged that the density of the polymer chains and the chain length are crucial factors for determining the properties of the brush. Glycopolymer brushes have been prepared for the specific binding of lectins [85,86]. Interestingly, enzymatic elongation of the polymer chains of the brush is carried out directly on the solid substrate to optimize lectin binding. Moreover, impedimetric studies of the glycopolymer brush-coated electrode revealed better sensitivity of shorter than longer brushes owing to the hindered accessibility of lectin inside the longer brushes. Thus, polymer brushes could have potential use as biosensor platforms. 

### 3.3. Metal Nanoparticle-Based Sensors

**Figure 5:**
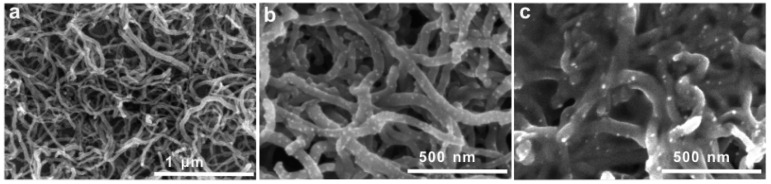
Scanning electron microscope images of (**a**) multi-walled carbon nanotubes (MWCNT), (**b**) Au nanoparticles-deposited MWCNT (Au/MWCNT); and (**c**) lectin-modified Au/MWCNT. Reprinted with permission from Zhang et al. [88].

Several groups have reported Au nanoparticle-based glycoprotein sensors prepared using different protocols. Au disk electrodes coated with alkanethiols, such as HS-(CH_2_)_2_-NH_2_, HS-(CH_2_)_6_-NH_2_ and HS-(CH_2_)_11_-NH_2_, were modified with Au nanoparticles layer through the affinity of the -NH_2_ group to Au. The Au nanoparticles were further modified with carboxyl-terminated alkanethiol, followed by immobilization of SNA lectin [87]. The SNA-modified sensors exhibit higher response to fetuin than asialofetuin and oxidized fetuin. The detection limit of fetuin is at attomolar levels. CNTs/Au nanoparticle composites are useful for the fabrication of lectin-based sensors for cancer cells [88]. CNTs are first deposited on the surface of a glassy carbon electrode by casting the CNTs dispersion (Figure 5a) and Au nanoparticles are electrochemically deposited on the CNTs-modified electrode from HAuCl_4_ solution (Figure 5b). The resulting electrode is covalently modified with SNA or Con A through thioglycolic acid as a linker (Figure 5c). The sensors thus prepared can detect glycan-bearing cells. To determine cell binding on the sensors, Au nanoparticles modified with a second lectin and thionine are used. The sensors afford voltammetric signals originating from thionine, which depend on the number of cells. Thus, cancer cells such as A549, H1299, and 95-D in the approximate concentration range of 10^4^–10^8^ cells mL^−1^ can be detected.

Au and Fe_3_O_4_ nanoparticles dispersed in polyvinylbutyral (PVB) have been used for the construction of impedimetric sensors for glycoproteins from patients infected by dengue [89,90]. The sensors are prepared by dip-coating the solution containing PVB, Au, or Fe_3_O_4_ nanoparticles and lectin. Sensors comprising Fe_3_O_4_ nanoparticles and CramoLL lectin exhibit impedimetric responses to fetuin and glycoproteins of dengue serum. The sensors show higher responses to dengue serotype 2 than to serotypes 1 and 3. Another group has also reported impedimetric sensors sensitive to dengue serotype 2 [91]. They used PANI/Au nanoparticle composites with electrostatically attached BmoLL lectin. Hemisphere electrodes bearing Au nanoparticles have also been used for constructing lectin biosensors for dengue virus [92]. 

## 4. PBA-Based Sensors for Glycoproteins

**Figure 6:**
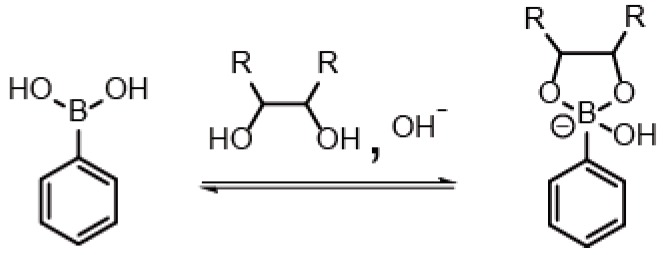
Binding equilibrium of PBA and diol compound.

PBA derivatives have been extensively studied as synthetic receptors for 1,2- and 1,3-diol compounds, such as sugars and catechols, because they selectively bind these compounds to form boronate esters [93]. Interestingly, boronate ester bonds of PBAs are cleavable to restore the original form of PBAs under acidic conditions, despite the covalent nature of the bonds (Figure 6). Thus, a variety of PBAs bearing chromophores and fluorophores have been synthesized for the development of optical sugar sensors, as well as sugar-sensitive materials [94,95,96,97]. In this context, it is anticipated that PBA-modified electrodes could serve as electrochemical sensors for glycoproteins and related compounds.

**Figure 7:**
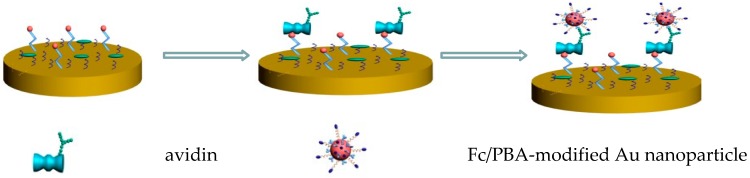
Avidin sensor based on Fc/PBA-modified Au nanoparticles. Reprinted from Xing et al. [99].

A PBA monolayer-modified Au electrode has been used as an extended gate for FET sensors for sialic acid and erythrocytes [98]. An Au electrode is covalently modified with PBA through amide linkages. The gate potential of the FET sensors changes as a result of erythrocytes binding through the sialic acid moieties of erythrocytes. In contrast, the sensor exhibits lower response to sialidase-treated erythrocytes, which contain a decreased amount of sialic acid, suggesting that the sensors could be used for monitoring the surface expression of sialic acid on erythrocytes. In addition, PBA-coated Au nanoparticles have been used to detect the glycoprotein avidin. For this purpose, Au nanoparticles are modified with PBA and ferrocene (Fc) simultaneously and coupled with biotin-modified electrodes (Figure 7) [99]. Avidin is a glycoprotein that exhibits extraordinary high affinity to biotin (binding constant, approximately 10^15^ M^−1^) [100]. In the Au nanoparticles, the Fc moiety affords redox signals while PBA groups serve as linkers to avidin. This sensor shows a linear response to 1.5–20 pM avidin with a lower detection limit of 0.2 pM. Note that PBA-modified electrodes are also useful for the construction of conventional enzyme sensors that is, enzyme-modified electrodes [101,102].

Various types of HbA1c sensors have been developed using PBA-modified electrodes. The choice of signaling mechanism is a key issue in the development of these sensors. GOx was used as a signaling protein in a competitive binding assay because it contains intrinsic hydrocarbon chains [103]. HbA1c and GOx competitively bind to the PBA-modified electrode, in which the sensor acquires an output signal through the GOx-catalyzed oxidation reaction of glucose. In fact, the oxidation current of hydrogen peroxide (H_2_O_2_) generated from the enzymatic reaction is monitored as an output signal. Fc-tagged anti-HbA1c antibody can be used as a redox marker in HbA1c sensors [104]. In this protocol, HBA1c is first captured on the surface of PBA-modified electrode and then the Fc-tagged antibody is attached to the captured HbA1c. The redox current originating from Fc moieties linearly depends on the concentration of HbA1c in the range of 5%–16%. Enzyme and redox labeling are not necessarily required for the construction of HbA1c sensors because HbA1cit possesses peroxidase-like catalytic activity. In practice, HbA1c sensors have been prepared using screen-printed carbon electrodes covered with a composite film of PBA-modified Au nanoparticles [105]. The catalytic current of the sensor is produced in the presence of H_2_O_2_. The sensor responds to 0.1%–1.5% HbA1c under optimum conditions. However, careful attention is required in this protocol because carbon-boron (C-B) bonds in PBA and its esters are sometimes oxidatively cleaved by H_2_O_2_ [106,107,108]. 

PBA-modified redox-active materials that change the electrochemical properties in response to HbA1c binding can be used for the preparation of label-free HbA1c sensors. Examples include PBA-bearing polyanilines nanoparticles (PBA-PANI) [109] and PBA-substituted pyrroloquinoline quinone (PBA-PQQ) [110]. PBA-PANI-modified sensors show oxidation peaks at 0.05 V in DPV and the peak current linearly depends on the logarithm of HbA1c concentration in the range 0.975–156 µM. The results can be explained by a HbA1c binding-induced ion flux blocking mechanism. On the other hand, PBA-PQQ-based sensors exhibit voltammetric signals at 0.1–0.2 V originating from the redox reaction of PQQ, which decreases upon binding HbA1c probably due to hindered electron transfer of PQQ. The percentage of HbA1c in blood samples from five healthy volunteers has been successfully determined with the sensor as 4.90%–5.86%. Impedimetric HbA1c sensors have also been prepared using interdigitated electrodes modified with thiopheneboronic acid [111,112,113].

An interesting protocol for the potentiometric determination of HbA1c has been studied in homogeneous solutions based on the selective complexation of PBA and alizarin red S (ARS) as a redox indicator [114]. The redox potential of ARS-PBA complex shifted from that of free ARS, while the original redox potential was restored upon the addition of HbA1c as a result of competitive binding of HbA1c to PBA. Based on the potential changes, the percentage of HbA1c in blood samples can be determined. This protocol would be more useful if the ARS-PBA complex could be immobilized in membranes, as in the case of polymer membrane-based potentiometric sensors [115,116]. In this regard, polymeric liquid membranes containing PBAs have recently been reported as potentiometric sensors for sugars [117,118].

A drawback of PBA-based biosensors lies in a lack of selectivity to specific glycoproteins because PBA exhibit a group selectivity to 1,2- and 1,3-diol compounds including sugars [93]. Therefore, it is not recommended to use PBA-based biosensors in the samples containing high concentration of sugars as contaminant. PBA-based sensors are nevertheless useful for discriminating glycoproteins from non-glycosylated proteins. In addition, it is necessary to take into consideration that the binding ability of PBAs is significantly pH-dependent. In most cases, PBAs show higher binding ability in neutral and weakly basic solutions than in acidic media because of the involvement of OH^−^ ion in the binding equilibrium of PBA and diol compounds (Figure 6). Thus, PBA-based sensors are usually used in neutral and weakly basic solutions. 

## 5. MIP-Based Sensors

MIPs are promising synthetic alternatives to antibodies. MIPs are prepared by the polymerization of monomers spatially-organized around a template molecule, followed by removal of the template from the resulting polymer. Therefore, monomers should have complementary binding sites to the template to produce MIPs with high selectivity [119,120]. Recent papers have summarized the synthesis of MIPs and their applications to bioanalysis [121,122,123]. In fact, several groups have employed molecular imprinting techniques for the development of glycoprotein sensors.

Electropolymerization of phenol in the presence of ovarian cancer marker CA125 affords polyphenol-based MIPs selective to CA125 [124]. The MIP-modified electrodes exhibit a redox current in a solution of Fe(CN)_6_^3−/4−^ ions, while the responses decrease upon the addition of target CA125 due to it blocking the cavity in the MIP. Molecular imprinting techniques are not limited to MIP; molecularly imprinted SAM can be prepared by forming SAM in the presence of target compounds. Based on this strategy, CEA-imprinted SAMs have been prepared on the surface of electrodes [125]. The electrode potential of the CEA-imprinted SAM electrode shifts in the presence of 2.5–250 ng·mL^−1^ of CEA, whereas no response is observed to hemoglobin as a non-target protein. The results are explained based on the selective binding of CEA to the cavity in the imprinted SAM. This type of potentiometric sensor would be useful because of its simplicity if the mechanism of the potential changes could be explicitly clarified. It is not always possible to prepare MIPs with high selectivity to proteins due to the characteristic features of proteins such as large molecular size, structural complexity, conformational flexibility, and limited solubility in organic solvents [126]. To improve the binding affinity and selectivity of MIPs, biological elements can be employed as additional components. For instance, a PSA-specific aptamer has been combined with a MIP in the construction of PSA sensors [127]. It was expected that the MIP cavity would synergistically bind PSA with the aptamer to enhance the binding specificity. The sensors show response to PSA in the concentration range 0.1–100 ng·mL^−1^, which is three-fold more sensitive than a PSA sensor without the MIP cavity. 

## 6. Conclusions

Electrochemical glycoprotein sensors have been developed based on metal and carbon electrodes modified with glycoprotein-binding materials including antibodies, lectins, PBA derivatives, MIPs, and so forth. Nanomaterials such as graphene, CNTs, and metal nanoparticles have recently been employed for developing high-performance sensors. The advantages of these nanomaterials in biosensor applications stem from the high surface area-to-weight ratio and easy modification of the surfaces. One of the problems to be solved in the future development of glycoprotein sensors is the design and construction of label-free and reagentless sensors for single-step measurements. Unfortunately, some of the glycoprotein sensors cited in this article require redox-active additives for signaling and others are based on multi-step measurements for the enhancement of signals. Further improvements in the design of the devices and materials would solve these problems.
